# Karyotype and genome size of *Iberochondrostoma almacai* (Teleostei, Cyprinidae) and comparison with the sister-species *I.lusitanicum*

**DOI:** 10.1590/S1415-47572009000200011

**Published:** 2009-06-01

**Authors:** Rita Monteiro, Cláudia Carvalho, Maria João Collares-Pereira

**Affiliations:** Centro de Biologia Ambiental, Departamento de Biologia Animal, Faculdade de Ciências, Universidade de Lisboa, LisboaPortugal

**Keywords:** Ag/CMA_3_-banding, flow cytometry, NORs, chromosome polymorphism, spontaneous triploidy

## Abstract

This study aimed to define the karyotype of the recently described Iberian endemic *Iberochondrostoma almacai*, to revisit the previously documented chromosome polymorphisms of its sister species *I.lusitanicum* using C-, Ag-/CMA_3_ and RE-banding, and to compare the two species genome sizes. A 2n = 50 karyotype (with the exception of a triploid *I.lusitanicum* specimen) and a corresponding haploid chromosome formula of 7M:15SM:3A (FN = 94) were found. Multiple NORs were observed in both species (in two submetacentric chromosome pairs, one of them clearly homologous) and a higher intra and interpopulational variability was evidenced in *I.lusitanicum*. Flow cytometry measurements of nuclear DNA content showed some significant differences in genome size both between and within species: the genome of *I. almacai* was smaller than that of *I.lusitanicum* (mean values 2.61 and 2.93 pg, respectively), which presented a clear interpopulational variability (mean values ranging from 2.72 to 3.00 pg). These data allowed the distinction of both taxa and confirmed the existence of two well differentiated groups within *I. lusitanicum*: one that includes the populations from the right bank of the Tejo and Samarra drainages, and another that reunites the southern populations. The peculiar differences between the two species, presently listed as “Critically Endangered”, reinforced the importance of this study for future conservation plans.

## Introduction

The new genus *Iberochondrostoma* was proposed by Robalo *et al.* (2007a) to accommodate some Iberian endemic arched-mouth nases, namely the recently described species *Chondrostomaalmacai* Coelho, Mesquita and Collares-Pereira, 2005 and its sister-species *Chondrostoma lusitanicum* Collares-Pereira, 1980*.* According to Coelho *et al.* (2005), *I*. *almacai* is confined to the southwestern Portuguese drainages of Mira, Arade and Bensafrim. Coelho *et al.* (2005) also revisited the morphological characterization and distribution of *I*. *lusitanicum*, since the populations of the new species were previously included within the *lusitanicum* geographic range.

**Figure 1:**
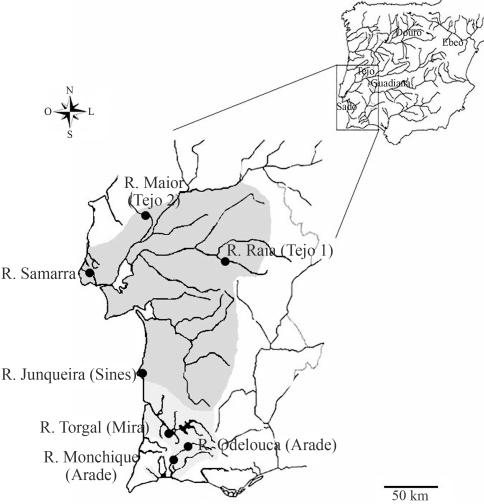
Sampling localities in each drainage and geographic distribution of *Iberochondrostoma lusitanicum* (dark grey - Samarra, Tejo and Sado drainages) and *Iberochondrostoma almacai* (light grey - Mira and Arade drainages).

Thus *Iberochondrostoma lusitanicum* is now known to be confined to the Tejo drainage (only to the tributaries close to the estuary - *e.g.* the rivers Trancão, Maior, Jamor, Lage and Ossos), to the Sado drainage, and to some small coastal Atlantic drainages (both north of Lisbon - Lizandro, Samarra and Colares - and south of Lisbon - from Lagoa de Albufeira to the Sines region including the Junqueira stream) (Figure 1). This species has a highly fragmented distribution and a high level of population subdivision (Alves and Coelho, 1994; Coelho *et al.*, 1997; Mesquita *et al.*, 2001; Robalo *et al.*, 2007b). A recent study using microsatellites and cytochrome *b* (mtDNA) sequences and covering all of the species range (a total of 212 specimens) was conducted. The aim of that study was to estimate the species genetic diversity, to characterize the population genetic structure and to infer its demographic history (Sousa *et al.*, 2008) in order to define the best conservation strategy (see also Robalo *et al.*, 2007b, which was based on a smaller widely distributed sampling, using a fragment of the cyt *b* gene and the nuclear beta-actin gene).

The karyotype of *I. lusitanicum* was first described by Collares-Pereira (1983), who analyzed specimens collected at the species type locality in the Sado drainage. Further studies were performed by Rodrigues and Collares-Pereira (1996) on specimens collected at the Samarra, Tejo and Mira drainages. These authors established the haploid karyotype formula of 7M:15SM:3A (FN = 94) and described a high polymorphism in the number (two and four) and size of the nucleolar organizing regions (NORs) using silver-staining (Ag) and chromomycin A_3_ (CMA_3_) staining. This polymorphism was later confirmed by fluorescence *in situ* hybridization (FISH) with a ribosomal DNA (rDNA) probe (Collares-Pereira and Ráb, 1999). Although all three populations shared this polymorphism, the presence of two NORs was much more common in the Mira specimens (now assigned to *I. almacai*) than the multiple NORs condition, which was more widespread in the remaining populations (Samarra and Tejo, now considered within the *I. lusitanicum* range). These observations raised the interest in revisiting such polymorphism while precisely defining the karyotype of the new species *I. almacai*.

Therefore, the present study included material from several populations of both species (some not previously sampled) aiming to: *i)* describe the karyotype of *I. almacai* and revisit the polymorphism of *I. lusitanicum* using C-, Ag-/CMA_3_- and restriction enzymes (RE)- banding; and *ii)* measure the nuclear DNA content of *I. almacai* and compare the genome size of both species. The peculiar allopatric distribution and differentiation pattern of the two species reinforced the importance of this approach for future conservation plans since they were listed as “Critically Endangered” in the national Red Data Book (Rogado *et al.*, 2005).

**Figure 2:**
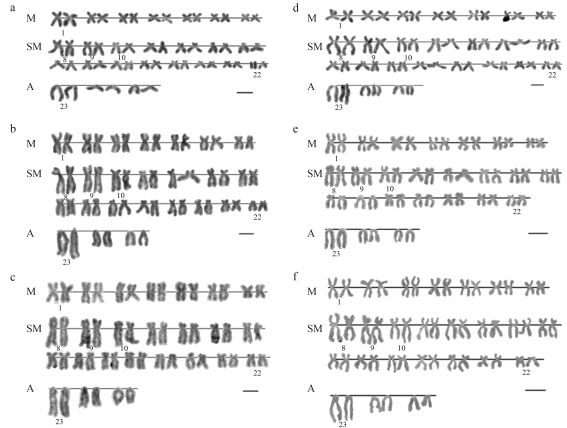
Karyotypes of specimens of *Iberochondrostoma lusitanicum* (*a-d*) and *I. almacai* (*e-f*) after RE-banding (1000x): *a*) Samarra - *Hae*III (female); *b*) Tejo2 - *Alu*I (female); *c*) Tejo1 - *Alu*I (female); *d*) Junqueira - *Hae*III (male); *e*) Odelouca *Alu*I (juvenile); *f*) Odelouca *Hinf*III (juvenile). The bar corresponds to 5 μm.

**Figure 3:**
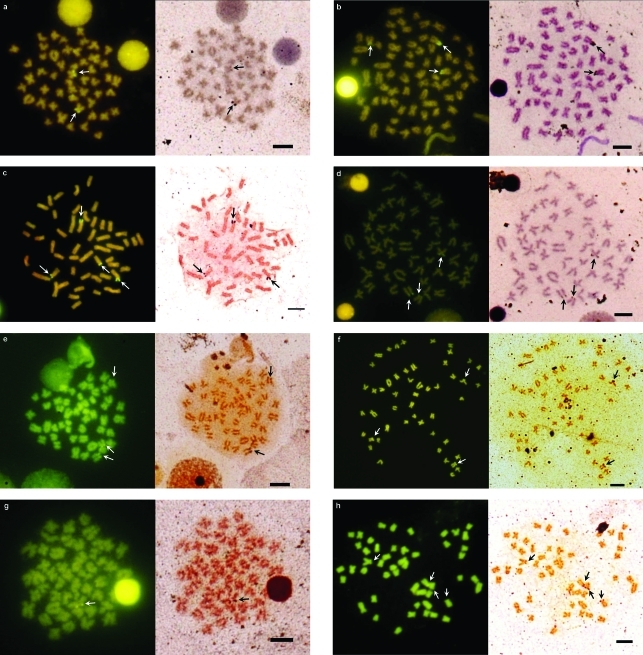
Sequential banding with CMA_3_/Ag in specimens of *Iberochondrostoma lusitanicum*: (*a*) Samarra, male, two CMA_3_/Ag-NORs; (*b*) Samarra, triploid female, three CMA_3_- and two Ag-NORs; (*c*) Tejo1, female, four CMA_3_- and three Ag-NORs; (*d*) Tejo2, female, three CMA_3_/Ag-NORs; (*e*) Junqueira, juvenile, three CMA_3_- and two Ag-NORs; Sequential banding with CMA_3_/Ag in specimens of *Iberochondrostoma almacai*: (*f*) Monchique, juvenile, three CMA_3_- and two Ag-NORs; (*g*) Odelouca, juvenile, one CMA_3_/Ag-NOR; (*h*) Torgal, juvenile, four CMA_3_/Ag-NORs. Arrows indicate the CMA_3_/Ag-NORs positive sites. The bar corresponds to 5 μm.

**Figure 4:**
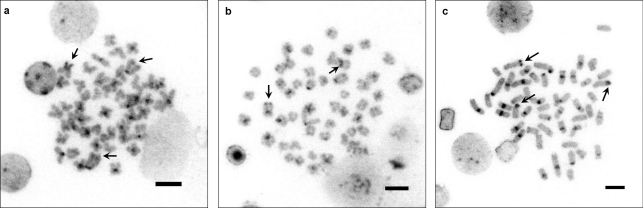
C-banding in metaphases of *Iberochondrostoma lusitanicum*: (*a*) Samarra, female, one pair of A chromosomes with terminal heterochromatin and one SM with a whole heterochromatic short arm; (*b*) Tejo2, male, one SM with a terminal block of heterochromatin and one SM with a whole heterochromatic short arm; (*c*) Tejo1, female, one pair of SM with terminal heterochromatin and one SM with a whole heterochromatic short arm. Arrows indicate the heterochromatic blocks. The bar corresponds to 5 μm.

**Figure 5:**
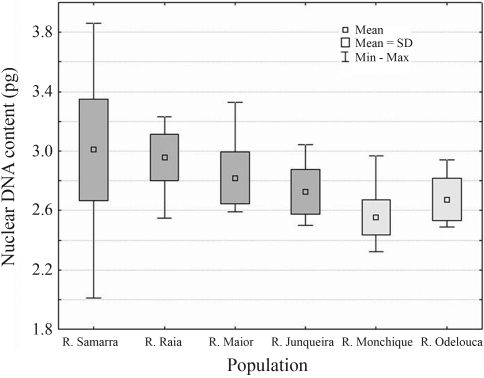
Mean values, ±SD and range of DNA content variation obtained by flow cytometry in the populations of *Iberochondrostomalusitanicum* (dark grey) and *I. almacai* (light grey).

## Material and Methods

The specimens examined are listed in Table 1 [s. Figure 1 for sampling sites - rivers Monchique and R. Odelouca (Arade drainage); Torgal (Mira drainage); Junqueira (Sines); Raia and Maior, two tributaries from the left and right banks of the Tejo drainage, respectively (named as Tejo1 and Tejo2) and Samarra]. Several collecting campaigns with seine nets and electrofishing were conducted from 2005-2007. Subsamples were transported each time to the laboratory and kept in an indoor aquarium for cytogenetic study. Fish were sacrificed with an overdose of the anaesthetic MS222 (Sandoz) in accordance to the recommended ethic guidelines (ASAB, 1998). All the specimens used for cytogenetic analysis were deposited at the collections of the Museu Nacional de História Natural (Museu Bocage, MB), Lisbon, Portugal (voucher numbers MB05-1924 through MB05-1936).

### Cytogenetic analysis

Chromosome preparations were obtained using the direct air-drying technique after *in vivo* (kidney cells) and *in vitro* (fibroblast fin culture) procedures (Rodrigues and Collares-Pereira, 1996). Fluorescent staining with the GC-specific chromomycin A_3_ (CMA_3_) was performed as described by Sola *et al.* (1992). Most of the metaphases were distained and sequentially examined in buffered Giemsa (5%, 7 min) and then Ag-stained using the method of Howell and Black (1980) with the modifications from Gold and Ellison (1982). C-banding and banding with restriction enzymes (RE - *Alu*I, *Hae*III and *Hinf*III) followed Jankun *et al.* (2004) with modifications. Slides were analyzed under an Olympus BX60 equipped with a DP50 Olympus digital camera. Karyotypes were arranged with the software UTHSCSA Image Tool version 3.0 and CorelDraw Suite 12. The chromosomes were arranged in a decreasing size order and classified according to their arm ratios (Levan *et al.*, 1964) in three morphological groups: metacentric (M), submetacentric to subtelocentric (SM) and acrocentric to telocentric (A). In order to establish the fundamental number (FN), the chromosomes of the M and SM groups were considered biarmed and those of group A were considered uniarmed.

### Genome size analysis

Blood samples were drawn from the caudal vein in all specimens (some in the field to allow their return to the river), mixed in buffer solution (40 mM citric acid trisodium salt, 0.25 M sucrose and 5% dimethyl sulfoxide) and immediately frozen at -80 °C. The ploidy of the fishes was determined by flow cytometry measurements of erythrocytes DNA content using an EPICS Profile II (Coulter) cytometer, following Collares-Pereira and Moreira da Costa (1999). Statistical analyses were performed with STATISTICA for Windows (version 7). The Tukey's multiple range test was used to test for significant heterogeneity in mean DNA content values between and within species (data not shown).

## Results

All the 26 specimens of *Iberochondrostoma almacai* analysed presented a diploid number of 50 chromosomes. The populations of both drainages (Arade and Mira) had a karyotype with FN = 94 composed of seven pairs of metacentrics (M), 15 pairs of submetacentrics/subtelocentrics (SM) and three pairs of acrocentrics/telocentrics (A) (Figure 2e-f). There was no evidence of sex chromosomes. After silver-staining, terminal Ag-NORs were frequently revealed in the short arms of the second submetacentric pair (pair 9, SM2) and more rarely also in one smaller submetacentric chromosome pair. Some population polymorphisms were also detected: one to three Ag-NORs (the maximum of three was only observed in two specimens) in the Arade samples and a maximum of two signals in the Mira population (Table 2). The number of NORs determined by CMA_3_-staining usually indicated an additional signal, *i.e.* usually three and more rarely four sites in specimens with three Ag-NORs. The number of NORs per cell also showed inter and intraindividual variation (Figure 3f-h).

Genome size measurements were only performed in Arade specimens (nine from Odelouca and 39 from Monchique) and did not yield significant differences. DNA contents ranged from 2.32 to 2.97 pg, with a mean value of 2.61 pg (SD = 0.13) for this species (Table 3).

All but one of the 86 specimens of *I. lusitanicum* analysed presented 2n = 50. One individual from the Samarra population was triploid with 3n = 75. The karyotypes were composed of seven pairs of metacentrics, 15 pairs of submetacentrics/subtelocentrics and three pairs of acrocentrics/telocentrics (FN = 94) (Figure 2a-d). The heterochromatic regions were mainly located in the centromeric regions with some exceptions: a distal block in the largest A pair and some small blocks in the two largest SM pairs on the Samarra specimens (Figure 4). Silver staining showed NORs in the terminal position in the short arms of the second submetacentric pair (SM2) and on a smaller submetacentric pair, *i.e.* two-three Ag-NORs (Table 2, Figure 3a-e), although the variation observed in all populations ranged from one to four (more rarely) signals. CMA_3_-staining also revealed one (very rare) to four positive signals, more frequently three-four with the exception of the Tejo1 (Raia) population which presented a much higher percentage of two NORs. The triploid specimen, a female with 99 mm of total length, exhibited two Ag- and three CMA_3_-NORs (Figure 3b). There was inter and intraindividual variation in the number of NORs per cell.

Genome size measurements gave a mean value of 2.93 pg (SD = 0.28) for the 152 specimens analysed, with a wider variation in the Samarra population (2.01-3.86 pg) after the exclusion of the triploid specimen (with 5.94 pg). This population mean DNA content was compared to the remaining ones, which ranged from 2.50-3.33 pg (Table 3 and Figure 5), and was significantly distinct from the Tejo2 and Junqueira mean values, as well as from the Tejo1 and Junqueira (data not shown).

## Discussion

The karyotypes of both sister species analysed were quite similar (2n = 50, 7M:15SM:3A, FN = 94). In this study we confirmed the presence of NORs in two chromosome pairs and observed that the polymorphism in the number of NORs previously described for *Iberochondrostoma lusitanicum* (Rodrigues and Collares-Pereira, 1996; Collares-Pereira and Ráb, 1999) also exists in *I. almacai,* although less pronounced*.* Moreover, the spontaneous occurrence of triploidy in *I. lusitanicum* was reported for the first time.

Our analyses evidenced that one pair of NORs had an identical location in the SM2 pair of both species. The chromosome pair bearing the second NOR could not be unequivocally identified without banding patterns because it is one of the 13 similar medium to small submetacentric pairs. Besides inter and intrapopulational variations in the number of NORs, some intraindividual differences were observed, which would not be expected unless independent structural changes occurred in individual cells (see also Galetti *et al.*, 1995).

NOR-phenotypes constitute useful karyotypic markers in fish cytotaxonomy and have been used for studying phylogenetic relationships among Cyprinidae (reviewed in Buth *et al.*, 1991 and Ráb and Collares-Pereira, 1995; but see *e.g.* Rábová *et al.*, 2003; Sola *et al.*, 2003; Bianco *et al.*, 2004; Gaffaroglu *et al.*, 2006; Ueda, 2007). Although NORs have been insufficiently analysed in Eurasian taxa, a single NOR-bearing chromosome pair is present in most studied Leuciscinae species and is considered a plesiomorphic character (revised by Ráb and Collares-Pereira, 1995 and Ráb *et al.*, 2007). Despite the apparent conservation observed in the subfamily, multiple NORs have been described in Eurasian and North American species and this condition was considered a derived character. In European species, multiple NORs were only reported in *Iberochondrostoma lusitanicum*, *Eupallasella perenurus* (Boron *et al.*, 1997; Boron, 2001), *Phoxinus phoxinus* (Boron, 2001) and more recently in *Achondrostoma oligolepis* (= *macrolepidotum)*, *Pseudochondrostoma duriense* (Gante *et al.*, 2004*),* and *Parachondrostoma arrigonis* (Kalous *et al.*, 2008).

Therefore, the species of the new Iberian *Chondrostoma* genera (Robalo *et al.*, 2007a), although apparently retaining the common leuciscine karyotype (eight pairs of M, 13-15 pairs of SM and two-four pairs of ST/A chromosomes and an ubiquitous medium-sized SM NOR-bearing pair, exhibited more differentiated NORs phenotypes and demand an in-depth investigation with molecular tools in a wider range of taxa (Ráb *et al.*, 2007). The present data allow hypothesizing that the common ancestral species to *I. almacai* and *I. lusitanicum* had multiple NORs. This phenotype might have been derived from the single pair of NORs through a chromosome translocation involving the rDNA region from the ancestral NOR-bearing Leuciscinae chromosome pair to another pair, as proposed by Collares-Pereira and Ráb (1999), prior to the speciation process. Moreover, population subdivisions may have favoured the stochastic fixation of multiple NORs during the differentiation process before the establishment of the drainages by the early Quaternary.

The mean genome size of *Iberochondrostomaalmacai* is statistically significantly smaller than that of *I. lusitanicum* (2.61 and 2.93 pg, respectively), which also presents a larger interpopulational variability, with mean values ranging from 2.72 to 3.00 pg (Figure 5). Collares-Pereira and Moreira da Costa (1999) defined a lower mean value (2.82 pg) based on only six fishes (five from Samarra and one from Tejo1). The wider variation found now (2.01-3.86) is likely due to the larger sampling of the Samarra population (N = 80), that showed a significant intrapopulational variability. Besides, fish from this population exhibited a comparatively larger genome size (mean 3.00 pg), significantly different from those of other *I. lusitanicum* populations. The DNA content of the triploid female, excluded from this analysis, is also hard to explain because its value approached that expected for a tetraploid specimen (5.94 pg).

Although our results indicated grossly similar chromosome morphologies for both species, *I. lusitanicum* had a higher number of Ag-NORs (three to four) than *I. almacai* (usually two and rarely three). A populational difference was also observed in *I. lusitanicum*: the populations from the northern part of the geographical range (the Samarra and Tejo drainages) were apparently distinct from the southern population (Junqueira/Sines) by their higher polymorphism and DNA content. This is in agreement with the differentiation pattern described by Sousa *et al.* (2008) who suggested the possible existence of four Evolutionary Significant Units (ESUs, sensu Moritz, 1994) - Samarra, north Tejo, south Tejo and Sado, Junqueira/Sines. However, since it was not possible to distinguish the role of ancient (related to drainage formation) from recent (anthropogenic-driven) events in the generation of the high genetic differentiation observed, Sousa *et al.* (2008) suggested that, in practical terms, at least two ESUs should be considered - the northern (Samarra and Tejo) and the southern (Sado and Junqueira/Sines) populations. Robalo *et al.* (2007b, 2008) did not include Junqueira/Sines and southern Tejo samples in their analyses, but instead sampled Lagoa de Albufeira (a small coastal lagoon between the Tejo and the Sado) and considered that the Lagoa and the Sado should be regarded as two independent ESUs. They also proposed that the populations from the northern Tejo and the small northern coastal drainages including Samarra were a single ESU. Indeed the pattern of differentiation of the *I. lusitanicum* populations remains unclear mainly south of the Tejo drainage. The analysis of multiple markers covering all of the species range and an accurate morphological characterization of all the populations would help to understand this pattern.

In the meantime, the recent population decrease evidenced by Sousa *et al.* (2008) and the patterns of genetic divergence observed for both *I. almacai* and *I. lusitanicum,* which can be related to geological events affecting the evolution and isolation of the river courses (revised in Coelho *et al.*, 2005; but see also Robalo *et al.*, 2008 and Sousa *et al.*, 2008), indicate that specific conservation measures are highly recommended. Populations of both species are subjected to seasonal fluctuations in their habitat, typical of Mediterranean type streams, with floods in winter and severe droughts in summer causing genetic bottlenecks that are likely to promote recurrent genetic drift events (Coelho *et al.*, 2005; Magalhães *et al.*, 2007; Robalo *et al.*, 2008). Therefore they become highly vulnerable to anthropogenic actions, reinforcing the importance of adopting specific strategies for habitat protection (in particular in the summer refugia) and eventually also for restocking programs. However, the source of donor specimens for such actions has to be carefully evaluated if maintaining the current genetic population structure is deemed an important conservation goal and translocations should be forbidden in order to avoid biodiversity loss.

## Figures and Tables

**Table 1:** Number of specimens from each population subjected to each kind of analysis.

	Population	Cytogenetic analysis				Genome size analysis
		2n	CMA_3_/Ag - staining	C-banding	RE-banding	
*Iberochondrostomalusitanicum*	Samarra	36	36	2	12	81
	Tejo1	21	19	2	9	30
	Tejo2	20	20	2	8	25
	Junqueira	9	9	-	-	17
	Total	86	84	6	29	152

*Iberochondrostomaalmacai*	Torgal	5	2	-	-	-
	Monchique	10	5	-	2	39
	Odelouca	11	5	-	2	9
	Total	26	12	-	4	54

2n - diploid number; CMA_3_- chromomycin A_3_; Ag- silver nitrate; RE - restriction enzyme.

**Table 2:** Variation (percentage of occurrence) in the number of CMA_3_- and Ag-positive NORs in *Iberochondrostoma* species.

	Population	N	Number of plates	CMA_3_ positive signals (%)					Ag positive signals (%)			
				1	2	3	4		1	2	3	4
*Iberochondrostoma lusitanicum*	Samarra	36	844	4.15	19.91	57.46	18.48		19.19	39.45	35.19	6.16
	Tejo1	19	410	0.73	42.44	28.29	28.54		20.73	45.37	25.61	8.29
	Tejo2	20	556	0.36	17.09	53.06	29.50		16.73	40.11	40.29	2.88
	Junqueira	9	214	0.80	7.00	46.80	45.40		15.30	39.70	37.60	7.40

*Iberochondrostomaalmacai*	Torgal	2	44	-	15.90	56.80	27.30		18.20	81.80	-	-
	Monchique	5	94	-	10.60	77.60	11.80		19.10	79.80	1.10	-
	Odelouca	5	100	-	-	88.00	12.00		19.00	80.00	1.00	-

N - number of specimens analyzed; CMA_3_- chromomycin A3; Ag- silver nitrate.

**Table 3:** Statistics of the genome size data obtained by flow cytometry for the distinct populations of *Iberochondrostoma almacai* and *I. lusitanicum* (after exclusion of the triploid specimen).

	Population	N	Nuclear DNA Content (pg)		
			Mean (SD)	Min-Max	SE
*Iberochondrostomalusitanicum*	Samarra	80	3.00 (0.33)	2.01-3.86	0.038
	Tejo1	30	2.95 (0.15)	2.55-3.23	0.028
	Tejo2	25	2.81 (0.17)	2.59-3.33	0.035
	Junqueira	17	2.72 (0.15)	2.50-3.04	0.036
	Total	152	2.93 (0.28)	2.01-3.86	0.023

*Iberochondrostomaalmacai*	Monchique	39	2.55 (0.12)	2.32-2.97	0.020
	Odelouca	9	2.67 (0.14)	2.49-2.94	0.040
	Total	48	2.61 (0.13)	2.41-2.96	0.030

N - number of specimens analyzed; SD - standard deviation - SD; Min-Max - minimum and maximum values; SE - standard error.
